# Identification of a Novel Allosteric Site at the M_5_ Muscarinic Acetylcholine Receptor

**DOI:** 10.1021/acschemneuro.1c00383

**Published:** 2021-08-05

**Authors:** Wessel A. C. Burger, Patrick R. Gentry, Alice E. Berizzi, Ziva Vuckovic, Emma T. van der Westhuizen, Geoff Thompson, Mahmuda Yeasmin, Craig W. Lindsley, Patrick M. Sexton, Christopher J. Langmead, Andrew B. Tobin, Arthur Christopoulos, Celine Valant, David M. Thal

**Affiliations:** Drug Discovery Biology, Monash Institute of Pharmaceutical Sciences, Monash University, Parkville, Victoria 3052, Australia; Department of Pharmacology, Warren Center for Neuroscience Drug Discovery and Department of Chemistry, Warren Center for Neuroscience Drug Discovery, Vanderbilt University, Nashville, Tennessee 37232, United States; Drug Discovery Biology and ARC Centre for Cryo-electron Microscopy of Membrane Proteins, Monash Institute of Pharmaceutical Sciences, Monash University, Parkville, Victoria 3052, Australia; Drug Discovery Biology, Monash Institute of Pharmaceutical Sciences, Monash University, Parkville, Victoria 3052, Australia; The Centre for Translational Pharmacology, Institute of Molecular, Cell and Systems Biology, College of Medical, Veterinary and Life Sciences, University of Glasgow, Glasgow G12 8QQ, United Kingdom; Drug Discovery Biology, Monash Institute of Pharmaceutical Sciences, Monash University, Parkville, Victoria 3052, Australia

**Keywords:** allosteric modulation, M_5_ muscarinic acetylcholine receptor, computational biology, mutagenesis, selectivity

## Abstract

The M_5_ muscarinic acetylcholine receptor (mAChR) has emerged as an exciting therapeutic target for the treatment of addiction and behavioral disorders. This has been in part due to promising preclinical studies with the M_5_ mAChR selective negative allosteric modulator (NAM), ML375. The binding site of ML375 remains unknown, however, making it difficult to develop improved M_5_ mAChR selective modulators. To determine the possible location of the ML375 binding site, we used radioligand binding and functional assays to show that ML375 does not interact with the well-characterized “common” mAChR allosteric site located in the receptor’s extracellular vestibule, nor a previously proposed second allosteric site recognized by the modulator, amiodarone. Molecular docking was used to predict potential allosteric sites within the transmembrane (TM) domain of the M_5_ mAChR. These predicted sites were assessed using M_5_−M_2_ mAChR receptor chimeras and further targeted with site-directed mutagenesis, which enabled the identification of a putative binding site for ML375 at the interface of TMs 2−4. Collectively, these results identify a third allosteric site at the M_5_ mAChR and highlight the ability of allosteric modulators to selectively target highly conserved proteins.

**Figure:**
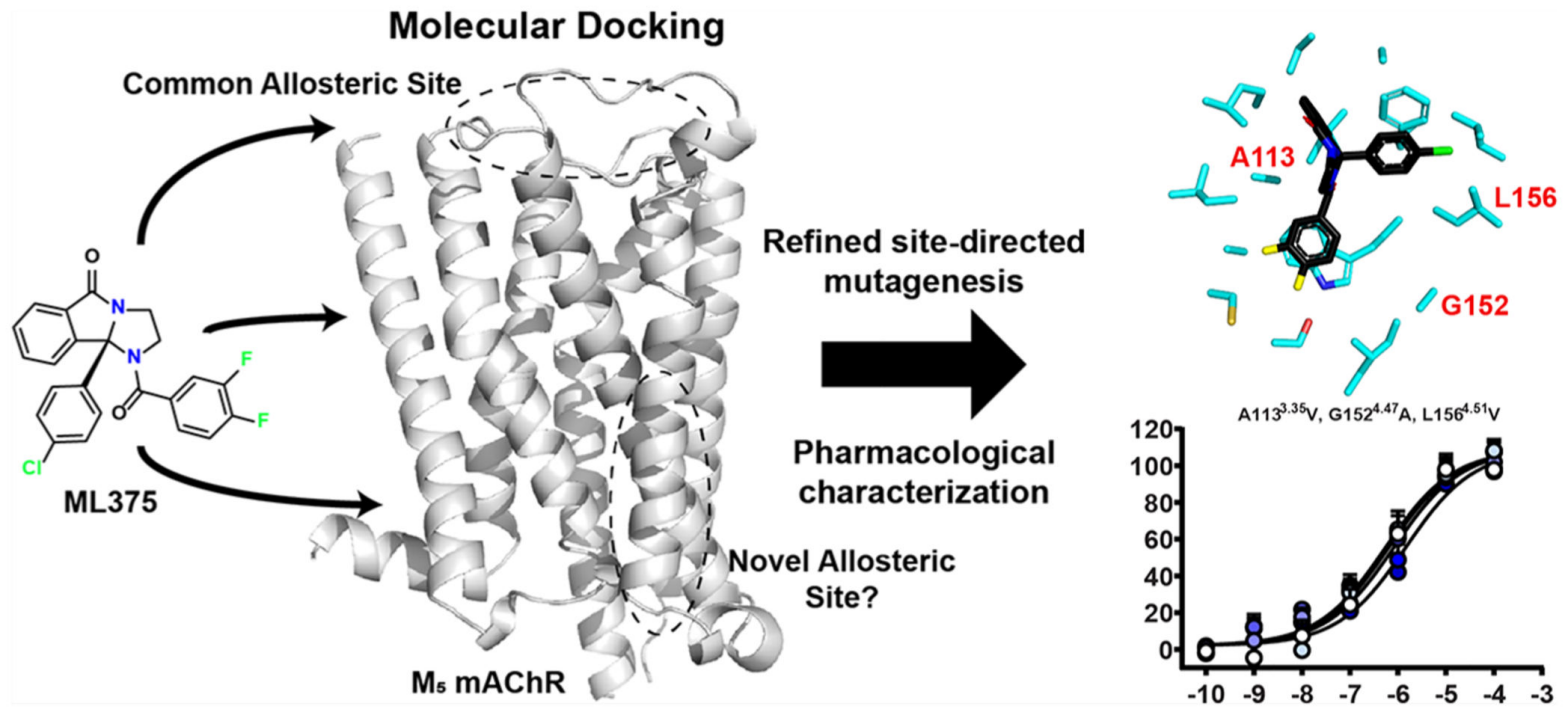

## Introduction

**Figure 1:**
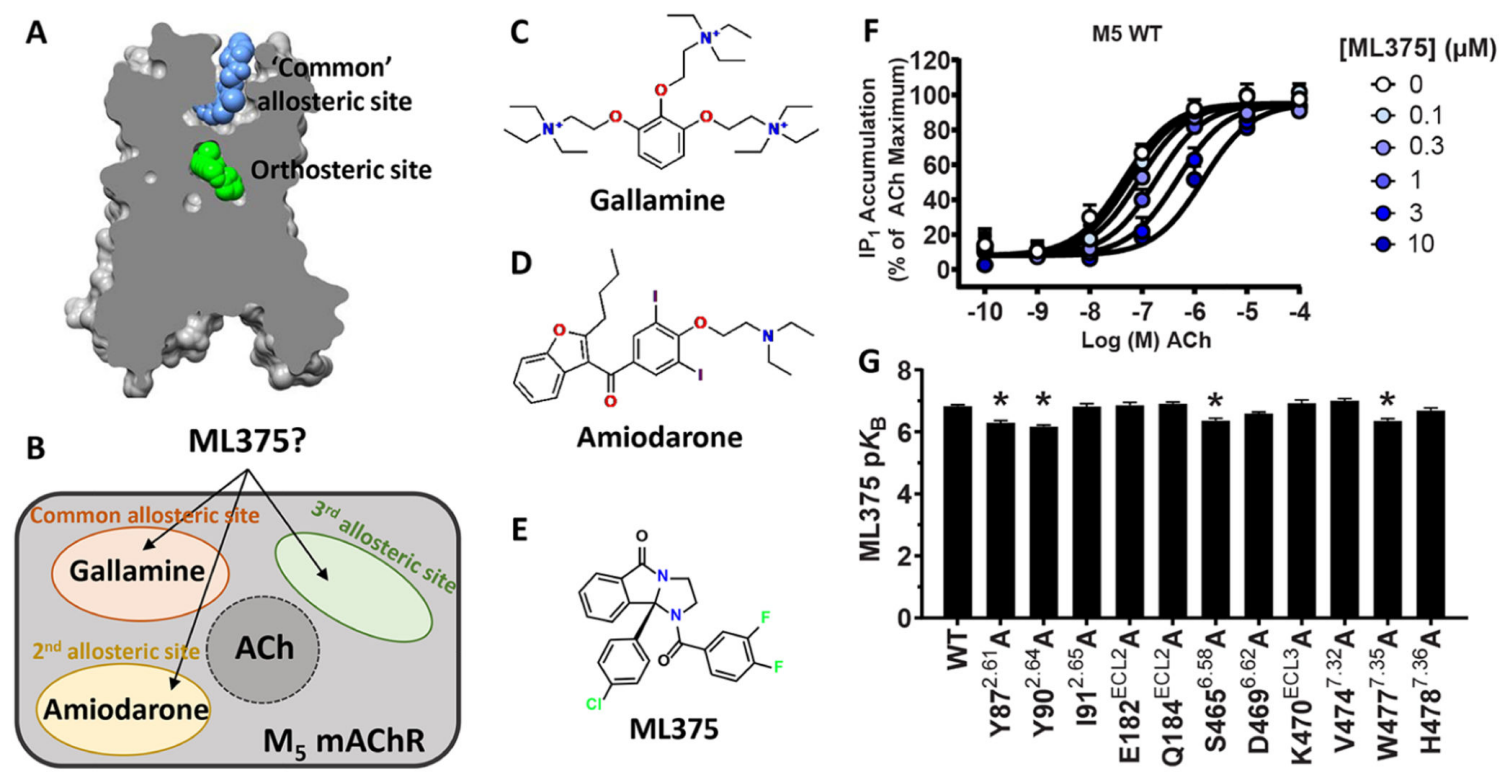
Allosteric modulators at the M_5_ mAChR. (A) Structure of the cobound LY2033298/iperoxo structure at the M_2_ mAChR highlighting the orthosteric site and the common ECV allosteric site (PDB: 4MQT). (B) Diagram of the potential ML375 allosteric sites at the M_5_ mAChR. (C) Prototypical M_2_ mAChR preferring NAM gallamine. (D) Atypical M_5_ mAChR modulator amiodarone. (E) M_5_ mAChR selective NAM ML375. (F) Interaction of ML375 with ACh in an IP1 accumulation assay in WT M_5_ mAChR-expressing CHO cells. (G) Effects of the M_5_ mAChR mutations on the p*K*_B_ of ML375. Data represent the mean ± SEM of 3 (mutants) or 12 (WT) independent experiments performed in duplicate. *, significantly different from WT, *p* < 0.05, one-way ANOVA, Dunnett’s post hoc test. Parameters obtained in these experiments are listed in Table 1.

The muscarinic acetylcholine receptor (mAChR) family consists of five subtypes (M_1_−M_5_) widely expressed throughout the human body. The M_1_, M_4_, and M_5_ mAChRs are primarily expressed in the central nervous system (CNS), while the M_2_ and M_3_ mAChRs are predominantly expressed in the periphery.^1^ Given the pivotal role of the M_1_ and M_4_ mAChRs in cholinergic signaling, they have long been considered attractive therapeutic targets for cognitive and psychotic disorders.^2^ Although the M_5_ mAChR makes up only 2% of the mAChRs expressed in the brain, it is exclusively expressed in regions that are involved in addiction, specifically, on dopamine-containing neurons of the substantia nigra pars compacta and the ventral tegmental area.^3–5^ Multiple studies have now confirmed a role for the M_5_ mAChR in regulating neuronal processes that contribute to alcohol, morphine, and cocaine addiction,^6–11^ as well as depression and anxiety.^12^ Unfortunately, the selective targeting of the M_5_ mAChR through the use of conventional orthosteric drugs has proven difficult, owing to the presence of a highly conserved orthosteric binding site across the mAChR family.^13^ One solution to this problem has been the development of ligands that target structurally distinct allosteric sites.^14^ Numerous studies have proposed at least one allosteric binding site, common to all mAChRs, located in an extracellular vestibule (ECV) positioned above the orthosteric binding site. The ECV is formed from residues in all three extracellular loops (ECL1−3) and extracellular portions of all seven transmembrane (TM1−7) helices^15–17^ (Figure 1A). The ECV has been pharmacologically characterized at all mAChRs using several prototypical allosteric modulators, such as the bis-ammonium alkane-type ligands C_7_/3-phth and W84, alcuronium, and gallamine (Figure 1B,C).^16,18–24^ Structural studies of the M_2_ mAChR bound to an allosteric modulator have further validated the ECV as a common allosteric site within mAChR subtypes.^17,25^ In addition, a second allosteric site has been proposed at the M_1_−M_4_ mAChRs on the basis of pharmacological studies with compounds such as KT 5720, WIN 62 577, WIN 51 708, and staurosporine.^26,27^ However, another allosteric site, recognized by the antiarrhythmic drug, amiodarone, and related derivatives, has also been proposed at the M_5_ mAChR^28^ (Figure 1D). In contrast to the common ECV allosteric site, the location(s) of these alternative M_5_ mAChR allosteric sites remain unknown. Excitingly, recent studies identified highly selective small molecule modulators of the M_5_ mAChR, exemplified by the negative allosteric modulator (NAM), ML375 (Figure 1E),^29^ that has preclinical efficacy in treating ethanol and opioid addiction,^8–11^ and the positive allosteric modulator with agonist activity (PAM-agonist), ML380.^30^ We recently demonstrated that the effects of both modulators potentially occur via interactions at a similar allosteric site on the receptor.^31^ Despite recent attempts to crystallize the M_5_ mAChR with ML375,^13^ the binding site for these selective M_5_ mAChR modulators remains unknown (Figure 1B). However, recent G protein-coupled receptor (GPCR) structures have revealed a breadth of allosteric sites across topographically distinct locations of GPCRs, including within the TM domain.^32^ The current study probed the binding site of ML375 through a combination of molecular docking and pharmacological interrogation of receptor mutants. We report that the mutagenesis of residues previously implicated in contributing to the common mAChR ECV allosteric site^15,17,21,22,33–36^ does not appreciably impact the activity of ML375. Additionally, the activity of ML375 was not altered by interaction with the well-known ECV modulator, gallamine, or a “second allosteric binding site” modulator, amiodarone. We subsequently combined molecular docking, chimeric receptors,^19,37^ and targeted mutagenesis to create “pocket specific” mutants that indicated an allosteric site for ML375 at the interface of TMs 2−4. Our data thus support the presence of a novel allosteric site on the M_5_ mAChR that adds to the growing number of allosteric sites now identified for multiple classes of GPCRs.^38^ The identification of this putative novel allosteric site will aid the further development of selective M_5_ mAChR allosteric modulators for the treatment of addiction and behavioral disorders.

## Results and Discussion

### ML375 Does Not Interact with the “Common” ECV Allosteric Site Residues at the M_5_ mAChR

The modulatory effect of ML375 at the wild-type (WT) M_5_ mAChR was determined in a G_q/11_-mediated inositol phosphate (IP) accumulation assay. In agreement with our previous study,^31^ ML375 behaved as a NAM of acetylcholine (ACh) at the WT M_5_ mAChR, with very high negative cooperativity such that it caused a concentration-dependent reduction in ACh potency that did not reach a limit (Figure 1F). Under such conditions, very high negative cooperativity (*α* → 0) is indistinguishable from a competitive interaction, thus allowing an application of the latter simpler model to the data to derive the affinity (p*K*_B_) of the allosteric modulator for its binding site.^39,40^ Accordingly, we analyzed the data using a Schild analysis.^41^ Upon application of this analysis, we found that the Schild slope was not significantly different to unity, again consistent with very high negative cooperativity, and its value was constrained to unity in order to derive a p*K*_B_ of 6.81 ± 0.07 for ML375 at the M_5_ mAChR.

**Figure 2:**
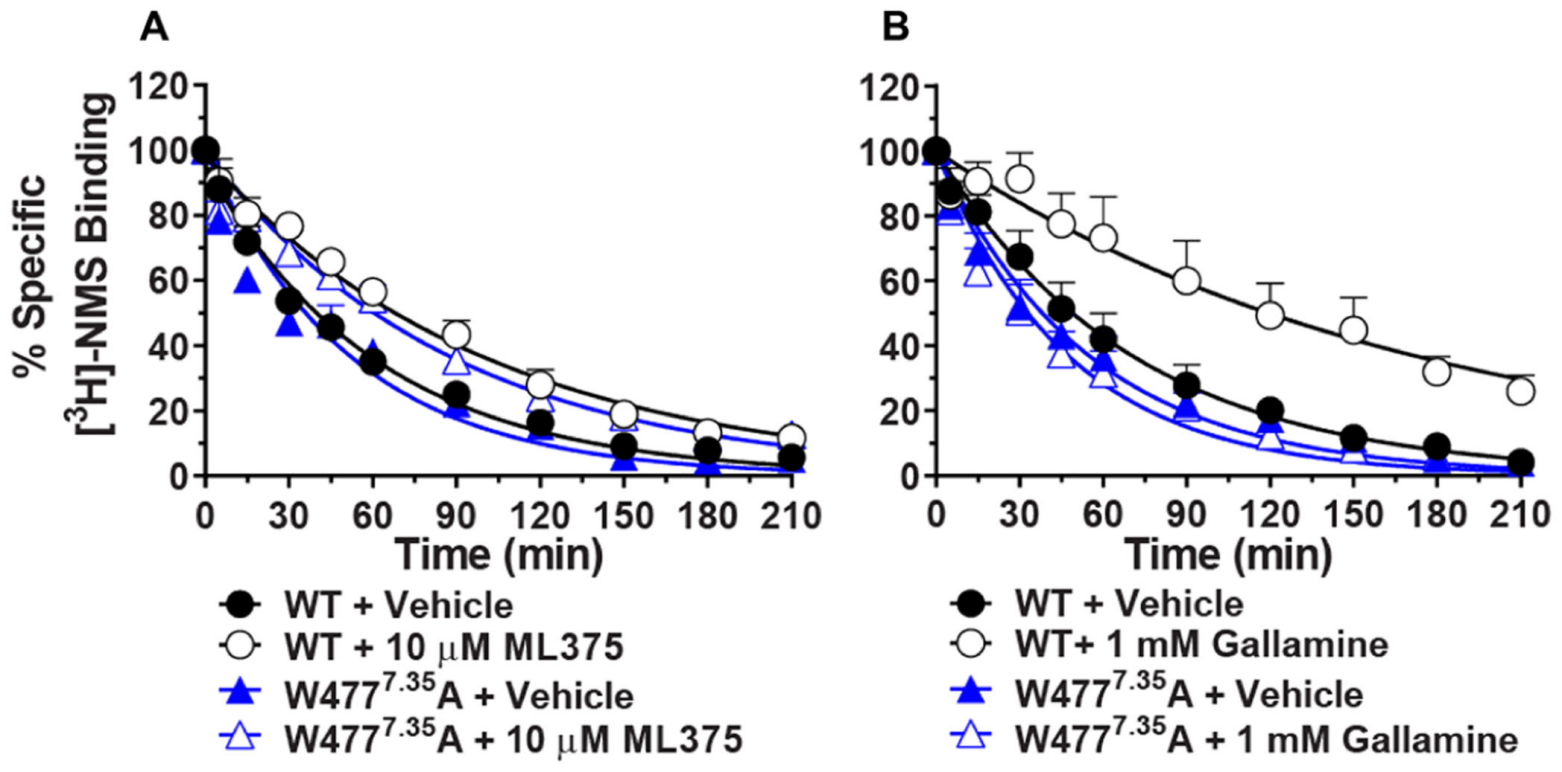
[^3^H]-NMS dissociation radioligand binding studies at the M_5_ mAChR. (A) The dissociation of [^3^H]-NMS was determined in the presence of vehicle (WT, *k*_off_ = 0.016 ± 0.001 min^−1^; W477^7.35^A, *k*_off_ = 0.017 ± 0.002 min^−1^) or 10 *μ*M ML375 (WT, *k*_off_ = 0.010 ± 0.001 min^−1^; W477^7.35^A, *k*_off_ = 0.011 ± 0.001 min^−1^). (B) The dissociation of [^3^H]-NMS was determined in the presence of vehicle (WT, *k*_off_ = 0.017 ± 0.002 min^−1^; W477^7.35^A, *k*_off_ = 0.020 ± 0.004 min^−1^) or 1 mM gallamine (WT, *k*_off_ = 0.004 ± 0.002 min^−1^; W477^7.35^A, *k*_off_ = 0.024 ± 0.005* min^−1^). Data points represent the mean ± SEM of 3 (A) or 4 (B) independent experiments performed in duplicate. *, significantly different from WT, *p* < 0.05, two-tailed Student’s *t* test.

The ECV of mAChRs is a well-established binding site for allosteric ligands that are commonly studied.^17,42^ Therefore, we mutated 11 residues in the M_5_ mAChR ECV to alanine to probe if ML375 interacts with the common mAChR allosteric site. These mutations were based on residues that were either nonconserved across the mAChR subfamily, previously identified to confer M_2_/M_5_ selectivity, or residues from previous studies that demonstrate their involvement in prototypical allosteric modulator binding at other mAChR subtypes.^15,17,21,22,33–36^ In general, the targeted mutations of the M_5_ mAChR exhibited little to no effect on the binding of ML375 quantified through ACh-mediated IP accumulation assays (Figure 1G; Table 1). A modest (~5-fold), albeit significant, decrease in ML375 affinity was observed at the Y87^2.61^A, Y90^2.64^A, S465^6.58^A, and W477^4.35^A mutants relative to WT (superscript numbering refers to the Ballesteros−Weinstein numbering scheme^43^). A change in ACh potency was also observed at these mutants, along with no significant changes in expression or [^3^H]-NMS binding (NMS, *N*-methylscopolamine), suggesting that these residues likely alter the conformational dynamics of the receptor instead of contributing directly to an allosteric binding site. No significant differences were observed at the remaining ECV mutants (Figure S1; Table 1). Previous work by Prilla et al. found that the W477^7.35^ residue in the M_5_ mAChR was vital to the binding of a number of structurally diverse prototypical allosteric modulators, including gallamine and multiple bis-ammonium allosteric ligands.^22^ To further explore the role of this residue in the binding of ML375, we performed dissociation kinetic experiments at the WT M_5_ mAChR and the W477^4.35^A mutant. At the WT, we found that ML375 significantly decreased the rate of dissociation of [^3^H]-NMS, confirming its allosteric mode of action (Figure 2A). Similarly, gallamine was able to slow down the dissociation rate of [^3^H]-NMS significantly (Figure 2B). Interestingly, the introduction of the W477^7.35^A mutation completely abolished the allosteric effect of gallamine but had no effect on ML375 (Figure 2A,B). Collectively, these mutagenesis studies confirm that gallamine binds to the common ECV allosteric site whereas ML375 likely binds elsewhere.

### Prototypical and Atypical Modulators Do Not Affect the Activity of ML375

**Figure 3:**
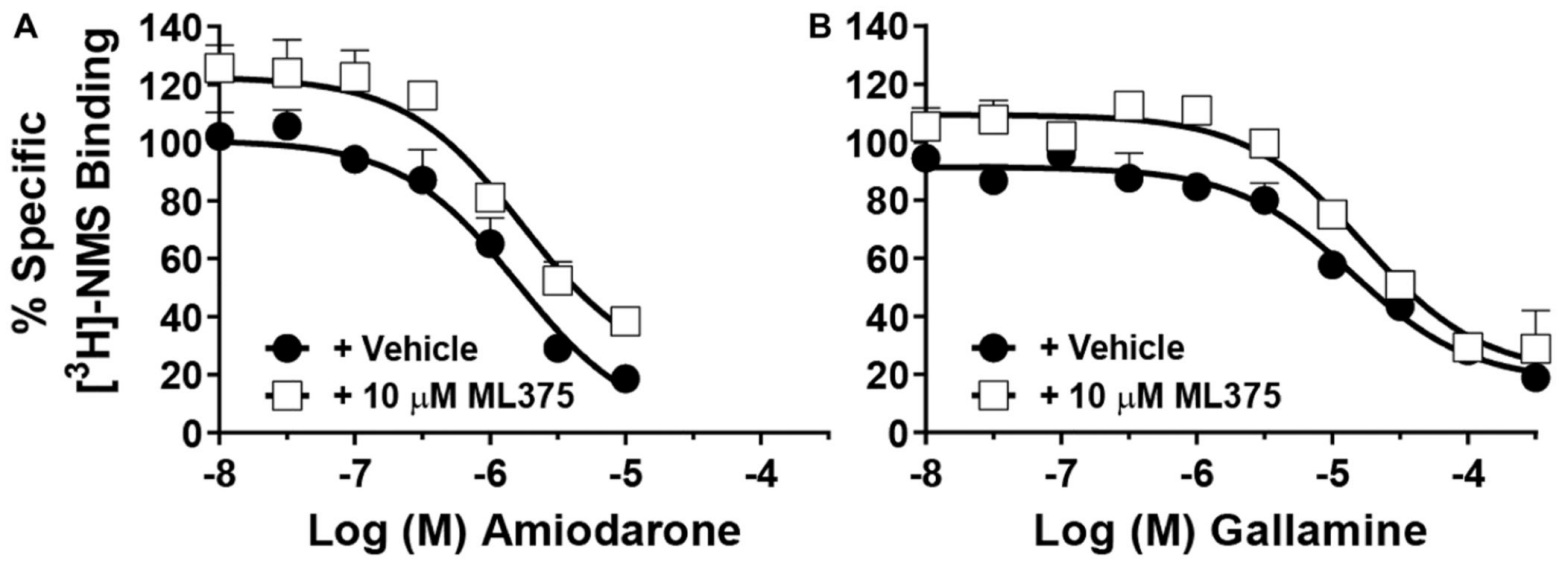
[^3^H]-NMS equilibrium radioligand binding studies at the M_5_ mAChR. (A) Competition binding curves of amiodarone in the absence (p*K*_B_ = 6.02 ± 0.13) and presence (p*K*_B_ = 5.97 ± 0.15) of 10 *μ*M ML375 in WT membranes. (B) Competition binding curves of gallamine in the absence (p*K*_B_ = 5.03 ± 0.11) and presence (p*K*_B_ = 4.96 ± 0.11) of 10 *μ*M ML375 in WT membranes. Data points represent the mean ± SEM of 3 independent experiments performed in duplicate.

In order to probe whether an interaction might occur between ML375 and a putative second mAChR allosteric site, we examined the effect of combining ML375 with amiodarone, as this ligand is hypothesized to bind to a yet-to-be-defined second allosteric site.^28^ We performed radioligand inhibition binding experiments with amiodarone, in the absence or presence of 10 *μ*M ML375, to establish whether ML375 could compete with amiodarone for the same allosteric site (Figure 3). In the absence of ML375, amiodarone had a p*K*_B_ of 6.02 ± 0.13. In the presence of ML375, a slight increase in [^3^H]-NMS binding was observed, consistent with ML375 displaying weak positive cooperativity with [^3^H]-NMS^29^ (Figure 3A); however, no significant effect on the affinity of amiodarone was observed [p*K*_B_ of 5.97 ± 0.15 (Figure 3A)], suggesting that amiodarone and ML375 are not competitive. Similarly, when interacting gallamine (p*K*_B_ = 5.03 ± 0.11) with ML375 (Figure 3B), the affinity of gallamine was unaltered (p*K*_B_ = 4.96 ± 0.11), consistent with our earlier findings (Figures 1G and 2), suggesting that ML375 does not compete for the gallamine allosteric site. Importantly, our pharmacological data suggest that gallamine, amiodarone, and ML375 bind to three distinct allosteric sites. Moreover, the lack of cooperative interactions in the binding assays also indicates that ML375 is a neutral allosteric ligand (NAL) with respect to both gallamine and amiodarone binding.

### Molecular Docking Suggests the Presence of Extrahelical Transmembrane Allosteric Sites

**Figure 4:**
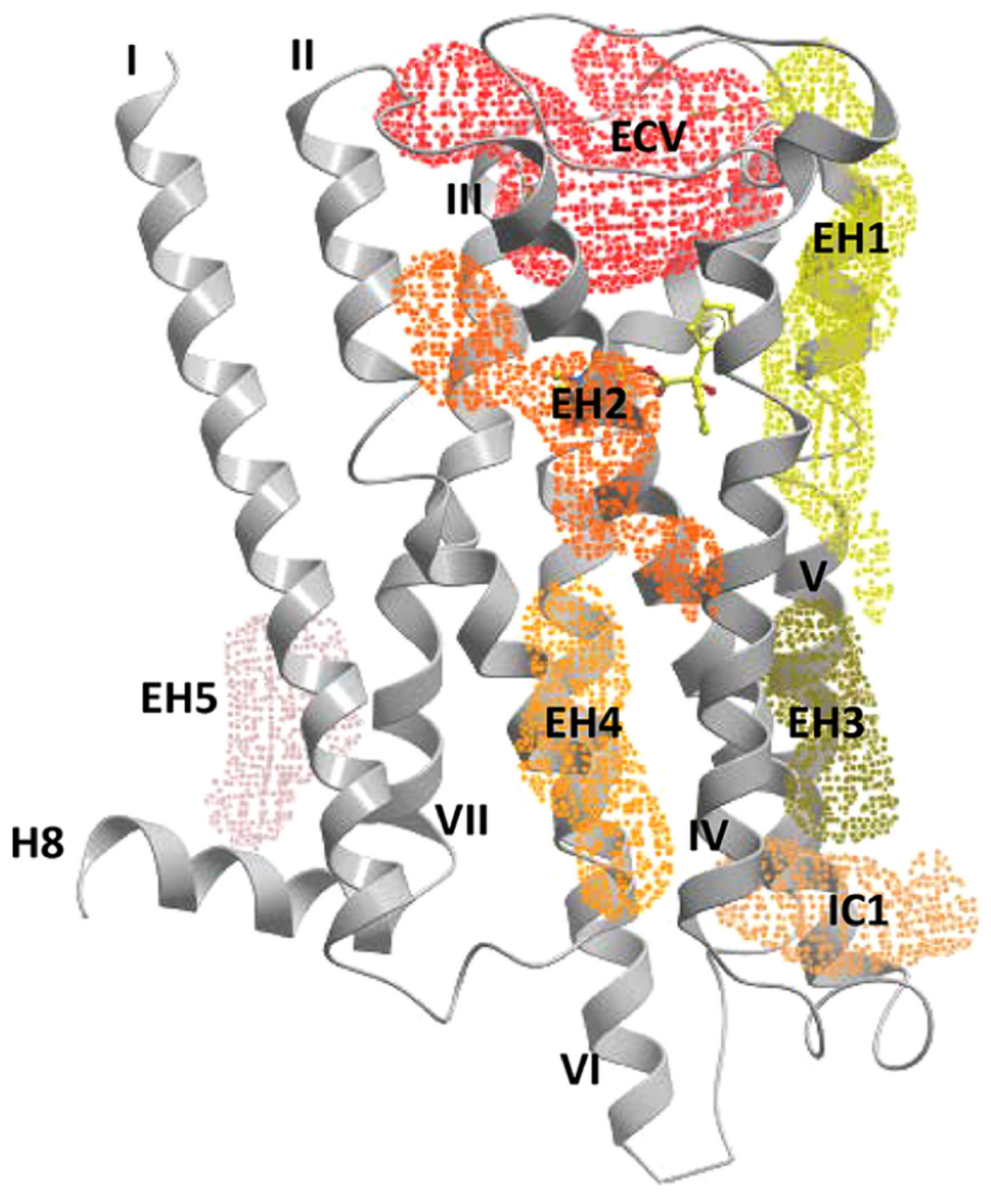
Location of pockets used for docking. The ICM Pocket Finder algorithm identified a pocket in the extracellular vestibule (ECV) and several extrahelical (EH) and intracellular (IC) pockets that were suitable for ligand docking. Shown in mesh are seven pockets across the surface of the M_5_ model (gray) used for the docking of ML375 into the receptor model. Tiotropium (yellow) is bound in the orthosteric pocket of M_5_ mAChR.

To further explore where ML375 might bind at the M_5_ mAChR, we used molecular docking. Numerous structural studies of GPCRs have revealed diverse locations for allosteric binding sites, including sites that are extrahelical, within or external to the 7TM bundle, or near the intracellular surface.^32^ Despite mAChRs serving as a long-standing model for studying allosteric modulation of GPCRs, the locations of additional sites have yet to be confirmed for this family of receptors. Therefore, we applied the Molsoft ICM pocket finder algorithm^44^ to our recently reported M_5_ mAChR crystal structure.^13^ Seven potential allosteric pockets were identified (Figure 4), and ML375 was docked into each pocket. Interestingly, the best scoring and most stable pose was with ML375 docked into the ECV (Figure S2A, ML375 in blue, Table S2) where it interacted with W477^7.35^, a residue that when mutated to alanine typically abolishes allosteric modulator activity^22^ but has no effect on ML375 (Figure 2A). In the crystal structure of the M_5_ mAChR, clear electron density was observed in the ECV that was attributed to a molecule of the crystallization buffer, PEG400^13^ (Figure S2A, PEG400 in orange), which was also previously observed in structures of the M_3_ and M_4_ mAChRs.^33,45^ To confirm that ML375 and PEG400 do not bind to the same site in the ECV, we performed kinetic dissociation experiments with [^3^H]-NMS, PEG400, and ML375. In the absence of ML375, increasing concentrations of PEG400 reduced the rate of [^3^H]-NMS dissociation, confirming an allosteric mode of action (Figure S2B). The addition of 10 *μ*M ML375, however, had no effect on PEG400’s ability to slow down [^3^H]-NMS dissociation, suggesting that they do not compete for the same ECV site (Figure S2C). To completely rule out the ECV as a binding site of ML375, we pharmacologically assessed full ECL1, ECL2, and/or ECL3 chimeric swaps between the M_5_ and M_2_ mAChRs that would alter a potential interaction site in the ECV. These constructs were recently used to study the effects of the ECL region on orthosteric and allosteric ligand binding at the M_5_ mAChR.^13^ Notably, there was no loss in ML375 affinity for any of the M_5_−M_2_ ECL chimeras or gain of ML375 affinity for any of the M_2_−M_5_ ECL chimeras (Figure S3), confirming that ML375 does not bind to an allosteric site in the ECV. The lack of an observed effect was not due to differences in orthosteric ligand binding, receptor function, or receptor expression (Table S1). The high molecular docking score of ML375 for the ECV site likely reflects the preference of docking algorithms for large solvent exposed cavities, such as the ECV, relative to small lipophilic intermembrane pockets.^46^ Outside of the ECV, ML375 was docked into five extrahelical (EH1−5) pockets as well as an intracellular pocket (IC1, Figure 4). Overall, the docking of ML375 gave reasonable binding scores at these TM pockets (Table S2). Considering the high lipophilicity of ML375 (cLogP = 5.2^47^), a binding site situated in the hydrophobic environment of the cell membrane or near the intracellular surface was a likely possibility.

### ML375 Engages with Residues within the Trans-membrane Domain of the M_5_ mAChR

**Figure 5:**
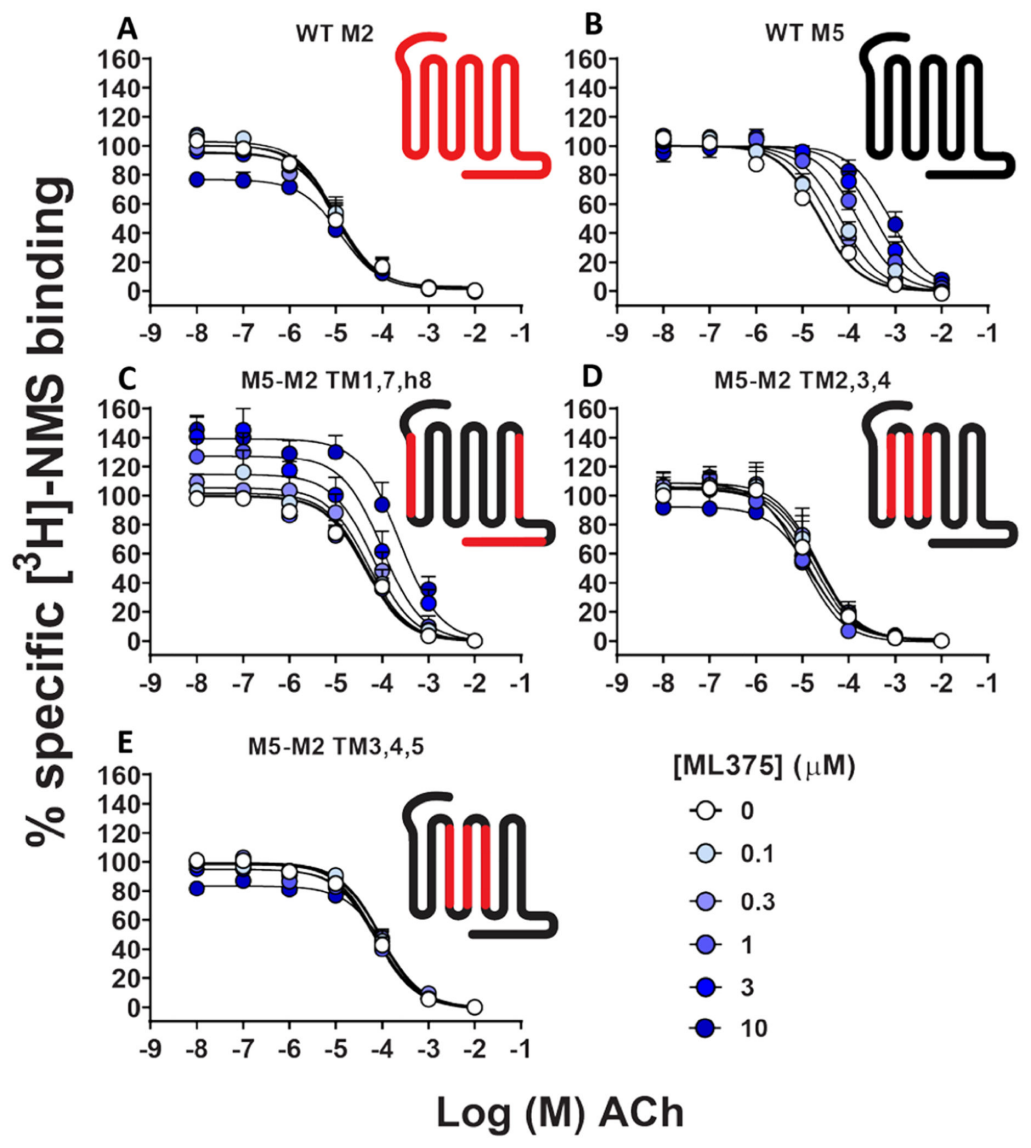
[^3^H]-NMS equilibrium radioligand binding studies at the M_2_ mAChR, M_5_ mAChR, and M_5_−M_2_ TM chimeric swaps. Interaction between [^3^H]-NMS, ACh, and ML375 in (A) WT M_2_, (B) WT M_5_, and (C−E) M_5_−M_2_ TM chimeric mutant-expressing CHO cells. The insets are cartoons of the WT M_2_, WT M_5_, and M_5_−M_2_ TM chimeras. Data points represent the mean ± SEM of 3−5 (mutants) or 4−12 (WT) independent experiments performed in duplicate. Parameters obtained in these experiments are listed in Table 2.

**Figure 6:**
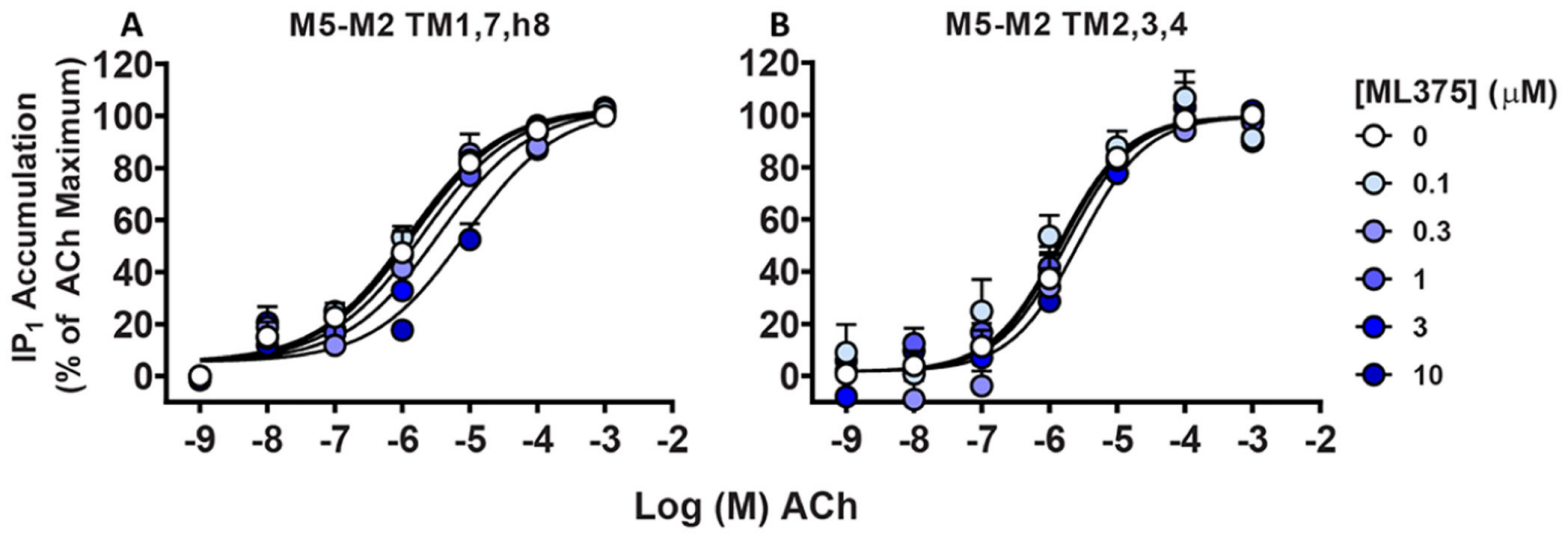
IP1 accumulation studies at the M_5_−M_2_ TM chimeric swaps. Interaction of ML375 and ACh in an IP1 accumulation assay in M_5_−M_2_ TM chimeric mutant-expressing CHO cells. Data represent the mean ± SEM of 5 independent experiments performed in duplicate. Parameters obtained in these experiments are listed in Table 2.

To probe whether ML375 might bind to any of the extrahelical or intracellular sites, we generated M_5_−M_2_ chimeras composed of multiple TM helices. Specifically, we generated M_5_−M_2_ chimeras of TM1, TM7, and helix 8 (covering the EH5 pocket); TM2, TM3, and TM4 (EH2 and EH4 pockets); and TM3, TM4, and TM5 swap (EH1, EH3, and IC1 pockets). These receptor constructs were stably transfected into CHO cells, and the affinity of ML375 and its binding cooperativity with ACh were quantified and compared to WT M_2_ mAChR and WT M_5_ mAChR in [^3^H]-NMS radioligand binding assays (Figure 5). Given the high selectivity of ML375 for the M_5_ mAChR over the M_2_ mAChR, we expected a loss of ML375 affinity for the M_5_−M_2_ TM chimeras, if ML375 was interacting with these regions. Swapping TM1, TM7, and H8 appeared to modestly reduce (~10-fold) ML375 affinity (Table 2). However, at the TM2,3,4 and TM3,4,5 chimeras, a complete loss in the modulation of ACh binding was observed, indicating that TMs 2−5 are important for the binding of ML375 (Figure 5D,E). To validate our findings, we performed IP accumulation assays with these three M_5_−M_2_ chimeras (Figure 6); however, only two of the three chimeras could be functionally investigated, M_5_−M_2_ TM1,7, H8, and TM2,3,4. At the M_5_−M_2_ TM3,4,5 chimera mutant, no IP accumulation response was observed due to the loss of Tyr217^5.62^ in this chimera mutant, a residue that is known to be crucial for the coupling of G_q_ at the M_1_, M_3_, and M_5_ mAChRs. By swapping this tyrosine residue to the equivalent residue at the M_2_ mAChR, a serine, a crucial interaction between TM5 and TM6 that enables G_q_ coupling is lost.^25,48,49^ Nonetheless, for TM1,7, H8, and TM2,3,4 chimeras, we were able to confirm a significant reduction and a complete loss of affinity for ML375, respectively (Table 2). Collectively, the pharmacological analysis of the chimeric receptors indicated that ML375 may bind to an allosteric site that is composed of residues belonging to TMs 2−5. Interestingly, similar allosteric sites involving the interface of TMs 2−4 have been identified at the cannabinoid 1 (CB1) receptor^50^ and at the interface of TMs 3−5 at the free fatty acid receptor (GPR40) and the C5a receptor 1 (C5aR)^51,52^ supporting the potential existence of a functionally important extrahelical allosteric binding site at the M_5_ mAChR.

### ML375 Binds to a Novel Allosteric Binding Site at the Interface of TMs 2−4

**Figure 7:**
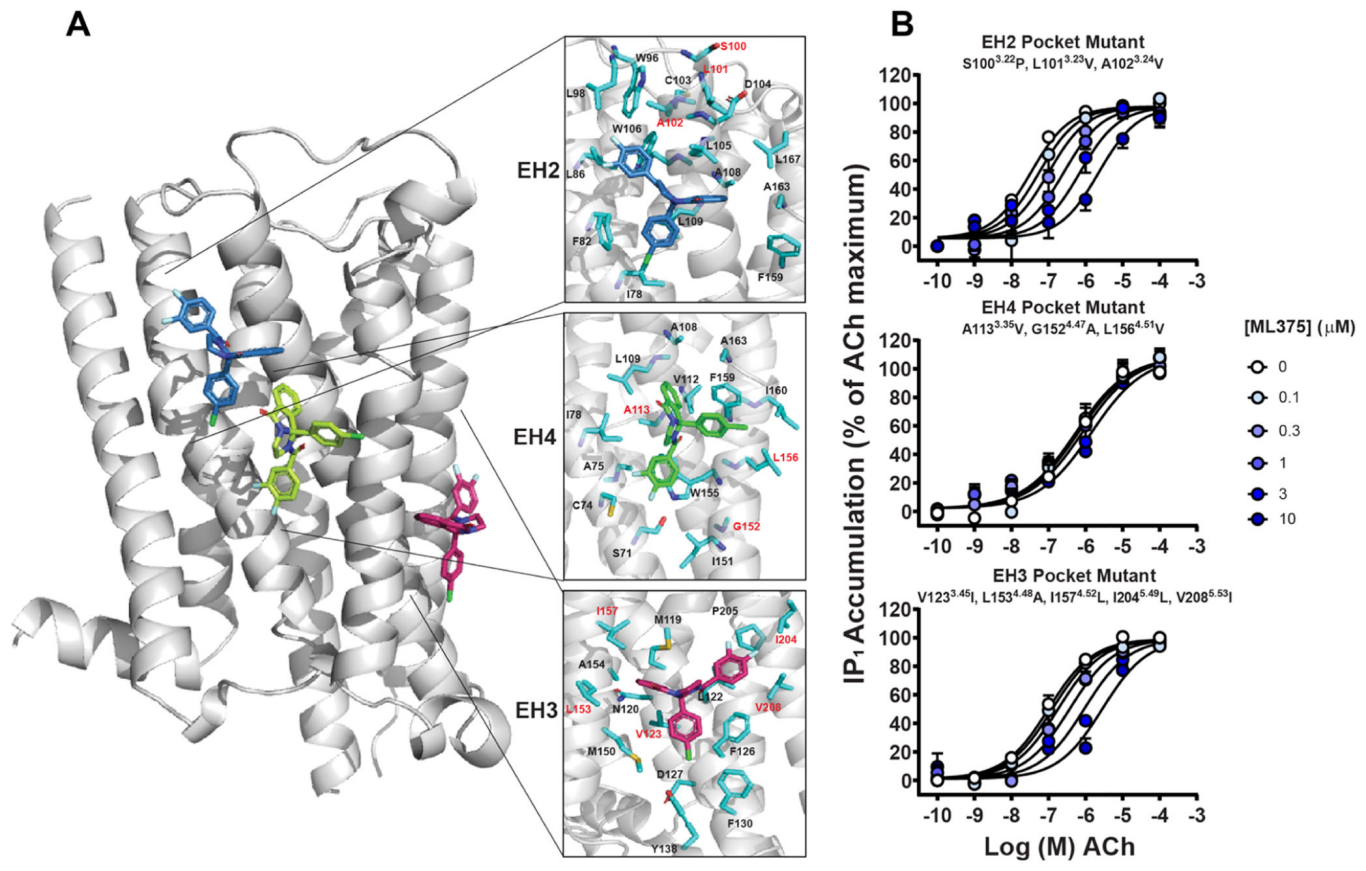
Identification of transmembrane allosteric pockets at the M_5_ mAChR. (A) Three potential pockets were identified that are made up of TMs 2−5. Shown in red are the residues that are nonconserved between the M_5_ and M_2_ mAChR. (B) Interaction of ML375 with ACh in an IP1 accumulation assay in M_5_−M_2_ pocket mutant-expressing CHO cells. Data represent the mean ± SEM of 3−4 independent experiments performed in duplicate. Parameters obtained in these experiments are listed in Table 2.

To more precisely define the ML375 binding site, we analyzed the allosteric pockets within TMs 2−5, which included the EH2, EH3, and EH4 pockets. These three pockets contain a number of residues each that are not conserved between the M_5_ and M_2_ mAChR (Figure 7A). Based on this, we designed pocket specific mutants where the nonconserved residues within each pocket were mutated to their M_2_ mAChR equivalent. All three pocket chimeras were stably transfected into CHO cells and tested in IP accumulation assays to assess and quantify the affinity of ML375 compared to the WT M_5_ mAChR. At the EH2 mutant and EH3 mutant receptors, ML375 was able to negatively modulate ACh function with an affinity similar to the WT M_5_ mAChR (Figure 7B and Table 2). Strikingly, at the EH4 mutant, ML375 had a significantly reduced affinity (p*K*_B_ = 5.21 ± 0.23) compared to the WT M_5_ mAChR indicating that the EH4 pocket represents a key part of the binding site for ML375 (Figure 7B and Table 2). To further validate EH4, the mutant was assessed in a radioligand binding inhibition assay (Figure S4 and Table 2). In line with IP accumulation assays, a significant decrease in both the affinity of ML375 and the ability of ML375 to modulate ACh binding was observed (Table 2). Collectively, these data suggest that the binding site of ML375 is located at the interface of TMs 2−4, with the nonconserved M_5_ mAChR residues of A113^3.35^, G152^4.47^, and L156^4.51^ playing an important role in the selectivity of ML375 and formation of this novel allosteric site. Inspection of this site in the crystal structure of the M_5_ mAChR^13^ reveals the tail end of a lipid molecule that binds between TMs 2−4 demonstrating that this site is clearly capable of binding lipophilic molecules.

Based on our data, it is intriguing to speculate on ML375’s mechanism of action, given its unique binding site. Molecular dynamics simulations have suggested that prototypical mAChR NAMs, such as the bis-ammonium alkane-type ligands, stabilize the inactive, open state of the M_2_ mAChR.^15^ Importantly, these simulations only investigated the “common” ECV allosteric site. When considering the location of the ML375 allosteric site, additional (or different) mechanisms may be required to produce an allosteric effect on the orthosteric site. Such mechanisms could take the form of those exhibited by the NAM, NDT9513727, at the C5aR, where the modulator stabilizes residues in TM3, TM5, and TM6 to prevent the conformational changes of TM5 and TM6 that are required for receptor activation.^51^ Alternatively, in the case of the NAM, ORG27569, at the CB1 receptor, the modulator inhibits the movement of TM6 that is required for receptor activation through stabilizing residues in TM2 and TM4.^50^ Notably, ORG27569 engages with residues C238^4.47^ and T242^4.51^, which are two of the nonconserved residues that were mutated in the EH4 pocket mutant at the M_5_ mAChR. The fact that the interaction between ML375 and ACh is characterized by high negative cooperativity, such that it is indistinguishable from a competitive interaction, indicates that ML375 inhibits the conformational changes required for ACh-mediated receptor activation. The most likely reason that this is not manifested as saturable and/or a noncompetitive reduction in the ACh maximal response (in addition to the reduction in ACh potency) is due to the high degree of stimulus-response amplification for the full agonist, ACh, in the IP accumulation assay. Our prior study using the weaker agonist, pilocarpine, against ML375 at the M_5_ mAChR showed a clear effect on both partial agonist potency and efficacy by the NAM.^31^ Irrespective, the demonstration of multiple NAM binding sites at the M_5_ mAChR, and across GPCRs in general, likely indicates that the mechanisms through which NAMs exert their effect will be in part influenced by their binding loci.

## Conclusion

Through the combined use of molecular docking with classical mutagenesis studies and extensive pharmacological characterization, we have identified a putative binding site for ML375, located at the interface of TMs 2−4. The ML375 binding site is at least the third, if not fourth, distinct allosteric site to be pharmacologically identified within the mAChR family, whereas it is only the second mAChR allosteric site for which a possible location has been identified. This highlights the fact that, although there is a wide assortment of allosteric binding pockets present in GPCRs, and likely within mAChRs, the identification of these sites in the absence of direct structural data is often a challenge. Ultimately, a ML375 bound M_5_ mAChR structure is needed to validate our putative ML375 binding site, as done in several recent studies confirming novel allosteric sites at other GPCRs.^32,53^ Unfortunately, such an approach with ML375 at the M_5_ mAChR proved unsuccessful.^13^ Despite this, the data presented herein are highly suggestive of ML375 binding to a site at the interface of TMs 2−4. The identification of a potential new allosteric site at the M_5_ mAChR within the TM domain presents new opportunities and new challenges for the design of selective allosteric modulators that could be used for the treatment of addiction and behavioral disorders. Unfortunately, ML375 has poor pharmacokinetics due to high plasma protein binding,^47^ and this highlights the physicochemical challenge for the generation of new allosteric compounds. Namely, these allosteric modulators must display sufficient lipophilicity to reach their site of action, while also displaying sufficient hydrophilicity to avoid being highly membrane and protein bound.

Future studies will investigate the potential existence of an equivalent “ML375” allosteric site at the M_1_−M_4_ mAChRs. The A113^3.35^ and G152^4.47^ residues are conserved at the M_1_, M_3_, and M_5_ mAChR subtypes, while the L156^4.51^ residue is conserved at the M_1_ mAChR subtype. At the remaining subtypes, this residue is a valine. This, in part, may explain why ML375 is selective for the M_5_ mAChR. Furthermore, it may also explain why a full loss in observable ML375 affinity occurred at the TM 2,3,4 and 5 M_5_−M_2_ chimeras, yet some activity was maintained at the EH4 pocket mutant. It is possible that the nonconserved residues of the EH3 pocket could play a role in determining the shape and size of the EH4 pocket, given that the EH3 pocket is parallel to the EH4 pocket. Therefore, the shape and size of the allosteric binding site may be more important to the selectivity of allosteric modulators at the M_5_ mAChR, in addition to the residue specific interactions.

It is expected that knowledge of the allosteric site of ML375 will aid the development of new-generation NAMs that are more suitable for use in human and rodent studies. Ultimately, it is expected the generation of new allosteric modulators for the M_5_ mAChR will increase the scope of modulating this promising therapeutic target.

## Methods

### Materials

DMEM and CHO FlpIn cells were purchased from Invitrogen (Waltham, MA). FBS was purchased from Thermotrace (Melbourne, Australia). Hygromycin B was purchased from Roche Applied Science. [^3^*H*]-*N*-Methylscopolamine ([^3^H]-NMS; specific activity, 70 Ci/mmol), UltimaGold, and Optiphase Supermix were purchased from PerkinElmer Life and Analytical Sciences (Waltham, MA). The IP-One Gq assay kit was purchased from Cisbio (Codolet, France). All other chemicals were purchased from Sigma Chemical Company (St. Louis, MO).

### Molecular Biology

The M_5_ mAChR DNA was cloned into a pEF5/FTR/V5 vector (Invitrogen, Waltham, MA) for the generation of stable cell lines using the Flp-In-CHO cell system (Invitrogen, Waltham, MA). Single M_5_ mAChR point mutations were made using site-directed mutagenesis with a single primer containing the desired mutation. To generate the M_5_−M_2_ TM chimeras, overlap extension PCR was used with primers specific to each TM region. All DNA constructs were sequenced to confirm the correct nucleotide sequence using the Australian Genome Research Facility (Melbourne, Australia).

### Generation of Cell Lines

DNA constructs in pEF5/FTR/V5 (Invitrogen, Waltham, MA) were stably expressed in FlpIn CHO cells (Invitrogen, Waltham, MA), which were maintained in high-glucose Dulbecco’s modified Eagle’s medium containing 10% FBS, 16 mM HEPES, and 600 *μ*g/mL hygromycin B. Mycoplasma testing was performed regularly on cell lines using the MycoAlertTM kit (Lonza, Basel, Swizerland); cell lines were mycoplasma-free before experiments were conducted.

### Preparation of CHO Cell Membranes

Cells were harvested with versene 48 h after subculture and centrifuged (300*g*, 5 min) before resuspension of the pellet in ice-cold phosphate buffer (50 mM Na_2_HPO_4_, pH 7.4). The cells were then homogenized with a Bio-Gen PRO200 homogenizer (3 × 10 s bursts with 30 s periods of cooling on ice between homogenizations) and centrifuged (300*g*, 5 min). The resulting supernatant was collected. The pellet was resuspended, and the homogenization and centrifugation process was repeated twice more, collecting the supernatant each time. The combined supernatant was then centrifuged at 30 000*g* for 30 min. The resulting pellet was resuspended and homogenized with a Polytron PT1200E homogenizer (3 × 10 s bursts with 30 s periods of cooling on ice between homogenizations) before use in the binding assays. Protein concentration was determined using the BCA assay with BSA as the standard.

### Equilibrium Binding Experiments

Constructs stably expressing the WT M_2_ mAChR, WT M_5_ mAChR, M_5_−M_2_ mAChR chimeras, and mutants were seeded in 96-well isoplates (PerkinElmer Life Sciences) at a concentration of 20 000−25 000 cells per well a day before the experiment was performed. These were incubated in a humidified atmosphere at 37 °C, 5% CO_2_ for at least 6 h. Whole cell experiments were performed in a final volume of 100 *μ*L with a HEPES-based buffer (110 mM NaCl, 5.4 mM KCl, 1.8 mM CaCl_2_, 1 mM MgSO_4_, 25 mM glucose, 50 mM HEPES, and 58 mM sucrose, pH 7.4). The affinity of [^3^H]-NMS for M_5_ mAChR receptor constructs was determined via saturation binding experiments where cells were incubated with a range of concentrations of [^3^H]-NMS. For interaction experiments, competition binding of a *K*_D_ concentration of [^3^H]-NMS by a range of ACh concentrations was performed in the presence of varying concentrations of ML375. For all experiments, nonspecific binding was determined in the presence of 10 *μ*M atropine, and total binding was determined in the presence of vehicle (0.1% DMSO). Bound radioactivity was assessed by liquid scintillation counting by a MicroBeta2 plate counter (PerkinElmer Life Sciences, Glen Waverley, Australia).

### Kinetic Experiments

#### Effect of Gallamine and ML375 on WT and W477^7.35^A Membranes

Cell membranes were initially preincubated with [^3^H]-NMS (final concentration of 1.2 nM) for 3 h at room temperature. Treatments containing atropine (final concentration of 10 *μ*M) and either gallamine (final concentration of 1 mM), ML375 (final concentration of 10 *μ*M), or vehicle (final concentration of 0.3% DMSO) were then added at time points spanning 3.5 h (5, 15, 30, 45, 60, 90, 120, 150, 180, and 210 min) to result in a final reaction volume of 1 mL. Nonspecific binding was determined in the presence of 10 *μ*M atropine, and total binding was defined as the 0 min time point. At the conclusion of the time course, the reactions were terminated in the manner described above.

#### Competition between PEG400 and ML375

Sf9 cells expressing M_5_-T4L (S117R) mAChR were harvested after 60 h. Sf9 cell membranes were prepared by homogenization and centrifugation. The final membrane pellet was resuspended in 20 mM HEPES pH 7.4 and 0.1 mM EDTA. Protein concentration was determined by absorbance at 280 nm, and membranes were stored at −80 °C. Assays were conducted in UniFilter-96 GF/B plates (PerkinElmer) with 1 *μ*g of membranes per well in a final volume of 300 *μ*L of binding buffer consisting of 20 mM HEPES, 100 mM NaCl, and 10 mM MgCl_2_ at pH 7.4. Membranes were initially preincubated with [^3^H]-NMS (final concentration of 1.2 nM) and a range of concentrations of PEG400 for 3 h at room temperature. Treatments containing atropine (final concentration of 10 *μ*M) and ML375 (final concentration of 10 *μ*M) or vehicle (final concentration of 0.3% DMSO) were then added at time points spanning 4 h (5, 10, 20, 40, 80, 180, and 240 min). Nonspecific binding was defined in the presence of 1 *μ*M atropine. Assays were stopped by vacuum filtration and washed three times with ice-cold 0.9% sodium chloride. Plates were allowed to dry before 40 *μ*L of Microscint-0 (PerkinElmer) was added to each well. Radioactivity was measured on a MicroBeta2 microplate counter.

### IP Accumulation Assay

Cells were seeded in 96-well plates at 10 000−25 000 cells/well (dependent on cell line) the day prior to the assay. The cells/well for each cell line was determined by the number of cells that gave an IP response within the linear range of the standard curve. On the day of the assay, media was exchanged for stimulation buffer (HBSS supplemented with 10 mM HEPES, 1.3 mM CaCl_2_, and 30 mM LiCl, pH 7.4) 60 min prior to stimulation with ligands. After 60 min, the buffer was replaced, and cells were then stimulated with ligands for 60 min at 37 °C and 5% CO_2_. Following 60 min of stimulation, drugs were removed, and the cells were lysed. Inositol phosphate (IP_1_) accumulation was then determined using the HTRF IP-One assay kit (Cisbio) with fluorescence measured using an EnVision plate reader (PerkinElmer).

### Computational Docking

Computational docking was done through use of the Molsoft ICM version 3.8-6 (Molsoft, LLC, San Diego, CA). The M_5_ mAChR crystal structure was used to generate a full atom receptor model through the molecular conversion procedure implemented in ICM molecular modeling software. The ICM Pocket Finder algorithm was used to predict binding pockets for the receptor model.^44^ ML375 was docked into the pockets through the in-built Multiple Receptor 4D Docking functionality and scored using the Biased Probability Monte Carlo (BPMC) global energy minimization.^54,55^

### Data Analysis

All statistical analyses and nonlinear regression curve fitting were done using GraphPad Prism (San Diego, CA).

IP1 accumulation assays measuring the functional interaction between the ML375 and ACh were characterized by high negative cooperativity that was indistinguishable from a competitive interaction (see the Results and Discussion section), and as such, the data were analyzed using the following classic competitive interaction model.^56^

(1) $$\text{response}\;=\;\text{bottom}\;+\frac{\left(E_{\text{max}}-\;\text{bottom}\;\right)}{1+{\left(\frac{10^{-{\text{pEC}}_{50}\left[1+{\left(\frac{\left(B\right)}{10^{-{\text{pA}}_{2}}}\right)}^{s}\right]}}{\left[A\right]}\right)}^{\text{HillSlope}\;}}$$

Here, pEC_50_ is the negative logarithm of the EC_50_ of ACh (*A*) in the absence of antagonist (*B*). HillSlope is the slope of the agonist curve, *S* is the Schild slope, and pA_2_ is the negative logarithm of the molar concentration of antagonist necessary to shift the agonist EC_50_ by a factor of 2. The Schild slope parameter, *S*, was not significantly different to unity as determined by an F-test and, as such, was constrained to 1, and therefore, the estimated pA_2_ values for each antagonist are equal to the p*K*_B_ (negative logarithm of the antagonist equilibrium dissociation constant).^56^

For radioligand saturation binding experiments with [^3^H]-NMS, total and nonspecific data were fitted to the following equation:

(2) $$Y=\frac{B_{\max}[A]}{[A]+K_{\text{A}}}+\text{NS}[A]$$

where *Y* is radioligand binding, *B*_max_ is the total number of receptors, [*A*] is the radioligand concentration, *K*_A_ is the equilibrium dissociation constant of the radioligand, and NS is the fraction of nonspecific radioligand binding.

Radioligand dissociation kinetics experiments were fitted to a monoexponential decay function.^56^ Inhibition radioligand binding curves between [^3^H]-NMS and unlabeled ligands were fitted to a one-site binding equation.^56^ IC_50_ values were converted to *K*_i_ values using the Cheng-Prusoff equation.^57^

All interaction radioligand-binding studies were analyzed according to the following adapted form of an allosteric ternary complex model that accounts for the interaction of two orthosteric ligands and one allosteric ligand on a receptor:^58^

(3) $$\begin{array}{l} Y=B_{\max}[A]/\{[A]+\left[\left(K_{\text{A}}K_{\text{B}}\right)/\left(\alpha_{\text{A}}[B]\right)+K_{\text{B}}\right]\\ \;\;\;\;\;\;\;\;\;[1+\text{(}[I]/K_{\text{I}}+\left([B]/K_{\text{B}}\right)+\left[\left(\alpha_{\text{I}}[I][B]/\left(K_{\text{I}}K_{\text{B}}\right)\right]\right]\text{\}} \end{array}$$

where [*A*], [*B*], and [*I*] represent the concentrations of the radioligand ([^3^H]-NMS), allosteric ligand, and orthosteric inhibitor, respectively; *K*_A_, *K*_B_, and *K*_I_ represent their respective equilibrium dissociation constants; and *B*_max_ is as defined above. The value *K*_A_ was fixed to the value determined from saturation binding experiments. The terms *α*_A_ and *α*_I_ represent the affinity cooperativity values between the allosteric ligand and the radioligand or orthosteric inhibitor, respectively; values greater than 1 indicate positive cooperativity; values <1 (but >0) negative cooperativity; and values of unity neutral cooperativity. All potency, affinity, and cooperativity parameters were estimated as logarithms.^59^ Where appropriate, fitted parameters were compared by an extra sum-of-squares F-test.^56^

## Supplementary Material

The Supporting Information is available free of charge at https://pubs.acs.org/doi/10.1021/acschemneuro.1c00383.

Interaction of ML375 and ACh in IP1 assays for all of the alanine mutations, ML375 and PEG400 radioligand dissociation experiments, interaction between [^3^H]-NMS and ML375 for all of the M_2_ and M_5_ ECL chimeras used in this study, ML375 binding at the EH4 pocket mutant, allosteric model parameters from radioligand binding assays for the interaction between ACh and ML375, and docking scores of ML375 (PDF)

Supporting Information

## Figures and Tables

**Table 1:** IP1 Accumulation and Saturation Binding Parameters for M_5_ mAChR Single Point Alanine Mutants^a^

mutant	[^3^H]-NMS saturation binding			IP1 accumulation for the interaction of ML375 vs ACh	
	p*K*_D_^b^	*B*_max_^c^(fmol/mg)		ACh pEC_50_^d^	ML375 p*K*_B_^e^
WT	8.78 ± 0.12 (4)	2522 ± 404 (4)		6.85 ± 0.04 (12)	6.81 ± 0.07 (12)
Y87^2.61^A	8.64 ± 0.06 (3)	2059 ± 99 (3)		6.49 ± 0.05^f^ (3)	6.30 ± 0.07^f^ (3)
Y90^2.64^A	8.38 ± 0.07 (3)	2076 ± 637 (3)		5.04 ± 0.04^*f*^ (3)	6.18 ± 0.05^*f*^ (3)
I91^2.65^A	8.29 ± 0.09 (3)	2705 ± 429 (3)		6.95 ± 0.07 (3)	6.82 ± 0.09 (3)
E182^ECL2^A	8.65 ± 0.12 (3)	2081 ± 354 (3)		7.02 ± 0.07 (3)	6.86 ± 0.08 (3)
Q184^ECL2^A	8.99 ± 0.05 (3)	2578 ± 392 (3)		6.78 ± 0.04 (3)	6.91 ± 0.05 (3)
S465^6.58^A	8.74 ± 0.09 (3)	1727 ± 651 (3)		7.16 ± 0.05^*f*^ (3)	6.37 ± 0.07^*f*^ (3)
D469^6.62^A	8.40 ± 0.08 (3)	3172 ± 196 (3)		7.01 ± 0.04 (3)	6.60 ± 0.05 (3)
K470^ECL3^A	8.88 ± 0.01 (3)	1973 ± 174 (3)		6.80 ± 0.10 (3)	6.92 ± 0.11 (3)
V474^7.32^A	8.81 ± 0.16 (3)	2236 ± 537 (3)		6.63 ± 0.07 (3)	7.00 ± 0.07 (3)
W477^7.35^A	8.62 ± 0.29 (3)	2143 ± 518 (3)		5.08 ± 0.05^*f*^ (3)	6.36 ± 0.06^*f*^ (3)
H478^7.36^A	8.46 ± 0.09 (3)	2480 ± 680 (3)		6.70 ± 0.07 (3)	6.69 ± 0.08 (3)

a Data represent the mean ± SEM of (*n*) independent experiments performed in duplicate.

b Negative logarithm of the radioligand equilibrium dissociation constant.

c Maximum density of binding sites.

d Negative logarithm of the concentration of ACh required to give the half maximal response.

e Negative logarithm of the antagonist dissociation constant.

f Significantly different from WT, *p* < 0.05, one-way ANOVA, Dunnett’s post hoc test.

**Table 2:** IP1 Accumulation and Binding Parameters for WT M_5_, WT M_2_, M_5_−M_2_ TM Chimeras, and Pocket Mutants^a^

constructs	[^3^H]-NMS saturation binding			interaction binding between [^3^H]-NMS and ACh in the presence of ML375					IP1 accumulation for the interaction of ML375 vs ACh	
	p*K*_D_^b^	*B*_max_^c^ (fmol/mg)		p*K*_i_ (ACh)^d^	log *α* (ACh)^e^	p*K*_B_^f^	log *α* (NMS)^g^		ACh pEC_50_^h^	P*K*_b_^i^
WT M_5_	9.12 ± 0.14 (4)	3517 ± 511 (4)		4.89 ± 0.14 (12)	−1.81 ± 0.02 (12)	6.79 ± 0.14 (12) N.D.	−0.001 ± 0.02 (12) N.D.		7.55 ± 0.08 (11) N.D.	6.45 ± 0.11 (11) N.D.
WT M_2_	9.91 ± 0.07 (4)	497 ± 109 (4)		5.43 ± 0.11 (6)	N.D.^j^	N.D.	N.D.		N.D.	N.D.
M_5_−M_2_ TM1,7,h8	8.84 ± 0.03 (4)	1114 ± 137^k^ (3)		4.47 ± 0.08 (5)	−3	5.59 ± 0.16^k^ (5)	0.23 ± 0.04^k^ (5)		5.90 ± 0.07^k^ (5)	5.77 ± 0.12^k^ (5)
M_5_−M_2_ TM2,3,4	9.23 ± 0.16 (4)	1254 ± 356^k^ (4)		5.00 ± 0.16 (4)	N.D.	N.D.	N.D.		5.84 ± 0.08^k^ (5)	4.98 ± 0.31^k^ (5)
M_5_−M_2_ TM3,4,5	9.23 ± 0.27 (3)	3457 ± 669^k^ (3)		4.27 ± 0.06* (5)	N.D.	N.D.	N.D.		N.R.^l^	N.R.
EH2 pocket mutant									7.47 ± 0.15 (4)	6.83 ± 0.18 (4)
EH3 pocket mutant									7.00 ± 0.09^k^ (4)	6.44 ± 0.12 (4)
EH4 pocket mutant	8.86 ± 0.11 (3)	1241 ± 342^k^ (3)		4.43 ± 0.06 (4)	−0.71 ± 0.2* (4)	5.72 ± 0.15* (4)	0.31 ± 0.05* (4)		6.28 ± 0.10^k^ (3)	5.21 ± 0.23^k^ (3)

a Data represent the mean ± SEM of (*n*) independent experiments performed in duplicate.

b Negative logarithm of the radioligand equilibrium dissociation constant.

c Maximum density of binding sites.

d Negative logarithm of the orthosteric agonist equilibrium dissociation constant.

e Logarithm of affinity cooperativity between the orthosteric agonist and allosteric modulator.

f Negative logarithm of the allosteric modulator equilibrium dissociation constant.

g Logarithm of the affinity cooperativity between [^3^H]-NMS and the allosteric modulator.

h Negative logarithm of the concentration of ACh required to give the half maximal response.

i Negative logarithm of the antagonist dissociation constant.

j N.D., not determined.

k Significantly different from WT M_5_, *p* < 0.05, one-way ANOVA, Dunnett’s post hoc test.

l N.R., no response.

## Abbreviations

ACh: acetylcholine
CNS: central nervous system
ECL: extracellular loop
ECV: extracellular vestibule
GPCR: G protein-coupled receptor
IP: inositol phosphate
mAChR: muscarinic acetylcholine receptor
NAL: neutral allosteric ligand
NAM: negative allosteric modulator
NMS: *N*-methylscopolamine
PAM: positive allosteric modulator
TM: transmembrane
WT: wild type
