# Supplementary material for: Identification of a Novel Allosteric Site at the M5 Muscarinic Acetylcholine Receptor
Source: ACS Chem Neurosci. Author manuscript; Available in PMC 2024 Jul 11. (PMC7616173; doi:10.1021/acschemneuro.1c00383)
Supplement: Supporting Information [file EMS197029-supplement-Supporting_Information.pdf]

Supporting Information

Identification of a novel allosteric site at the M_5 muscarinic acetylcholine receptor

Wessel A. C. Burger[1,#], Patrick R. Gentry[1,#], Alice E. Berizzi[1], Ziva Vuckovic[1], Emma T. van der Westhuizen[1], Geoff Thompson[1], Mahmuda Yeasmin[1], Craig W. Lindsley[2,3], Patrick M. Sexton[1,4], Christopher J. Langmead[1], Andrew B. Tobin[5], Arthur Christopoulos[1,*], Celine Valant[1,*] and David M. Thal[1,*]

[#] These authors have contributed equally.
*** Corresponding author.**

Author Contact Information
1. Drug Discovery Biology, Monash Institute of Pharmaceutical Sciences, Monash University, Parkville, Victoria, 3052, Australia.
2. Department of Pharmacology, Warren Center for Neuroscience Drug Discovery, Vanderbilt University, Nashville, TN 37232, USA.
3. Department of Chemistry, Warren Center for Neuroscience Drug Discovery, Vanderbilt University, Nashville, TN 37232, USA.
4. ARC Centre for Cryo-electron Microscopy of Membrane Proteins, Monash Institute of Pharmaceutical Sciences, Monash University, Parkville, Victoria, 3052, Australia.
5. The Centre for Translational Pharmacology, Institute of Molecular, Cell and Systems Biology, College of Medical, Veterinary and Life Sciences, University of Glasgow, Glasgow G12 8QQ, UK.

Corresponding Authors: Arthur Christopoulos (email: arthur.christopoulos@monash.edu), Celine Valant, (email: celine.valant@monash.edu) and David M. Thal (email: david.thal@monash.edu)

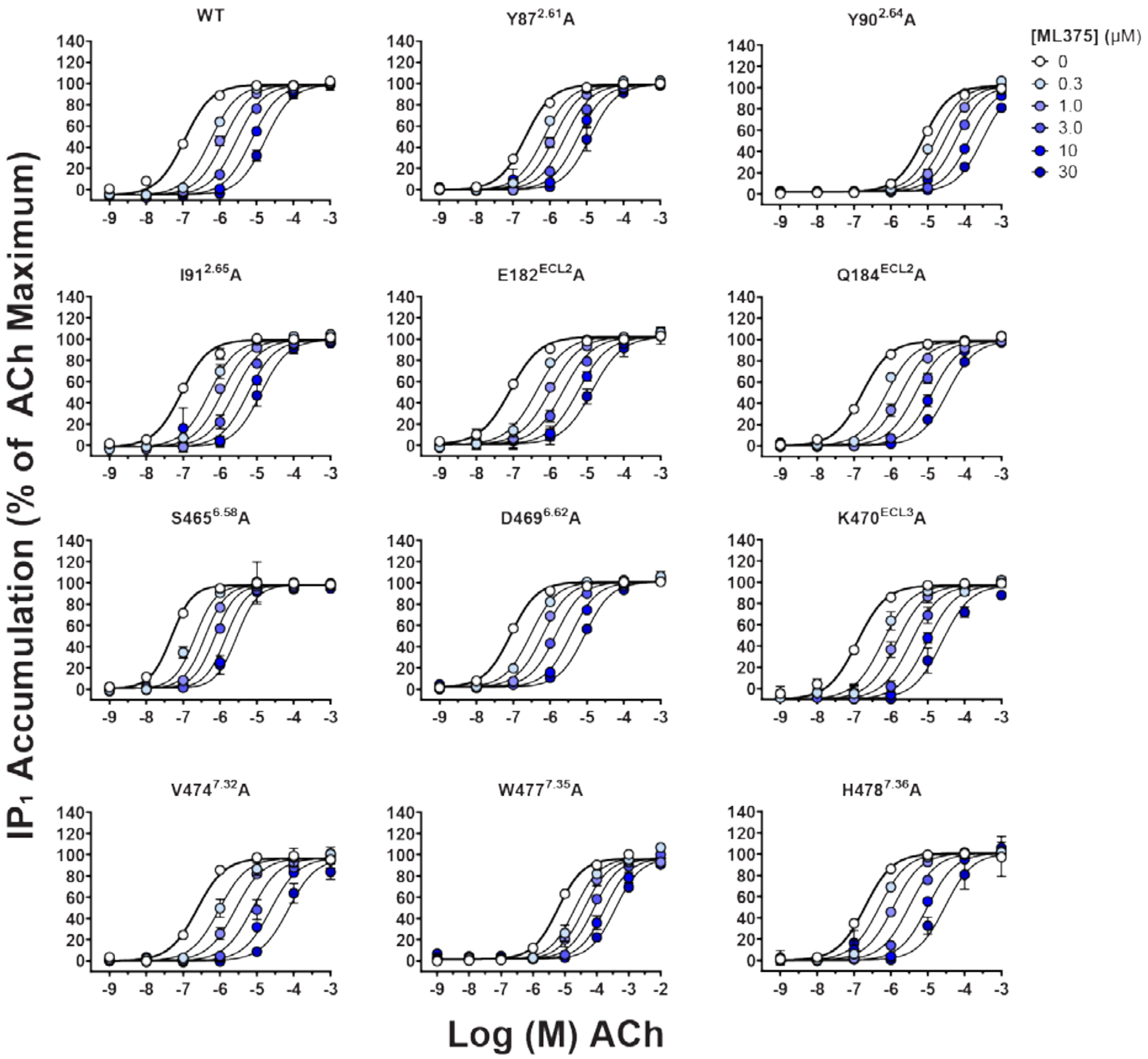

Supplementary Figure S1. Interaction of ML375 and ACh in IP1 assays for all of the alanine mutations. Data represent the mean ± S.E.M. of 3 (mutants) or 12 (WT) independent experiments performed in duplicate. Parameters obtained in these experiments are listed in Table 1.

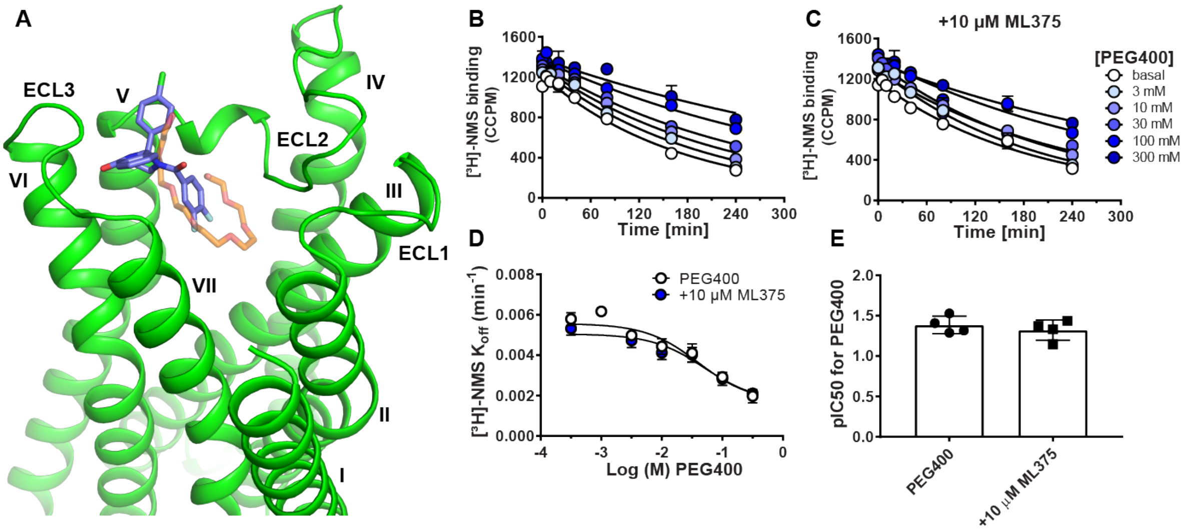

Supplementary Figure S2. ML375 does not compete with PEG400. (A) ML375 repeatedly docks into "common" ECV allosteric site, the same site where PEG400 was found in the recent M5 mAChR crystal structure (PDB: 6OL9, ML375 coloured blue, PEG400 orange). (B) [^{3}H]-NMS dissociation kinetics in the presence of increasing concentration of PEG400 and **(**C) in the presence of 10 µM ML375. (D) [^{3}H]-NMS dissociation rate constants are plotted against the PEG400 concentration to determine an apparent affinity (E) of approximately 40 mM for PEG400 in the absence and presence of 10 µM ML375. Experiments were performed on M_5-T4L(S117R) Sf9 cell membranes. Data points represent the mean ± S.E.M. of 3 independent experiments performed in duplicate. *, significantly different from WT, $p < 0.05$, two-tailed Student's *t* test.

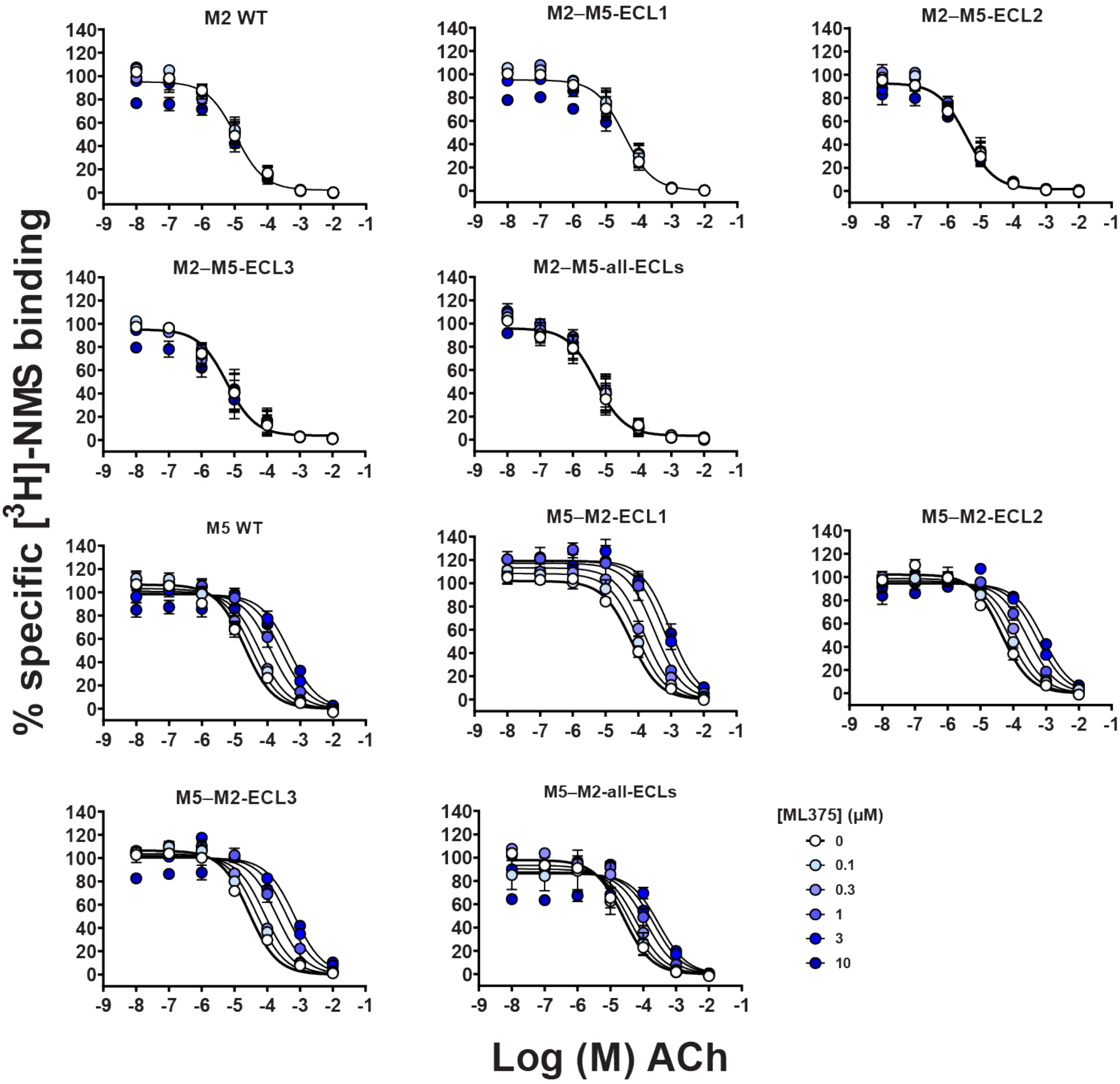

Supplementary Figure S3. Interaction between [^{3}H]-NMS and ML375 for all of the M_2 and M_5 ECL chimeras used in this study. Data represent the mean ± S.E.M. of 3-5 (mutants) or 4-12 (WT) independent experiments performed in duplicate. Parameters obtained in these experiments are listed in Supplementary Table 1.

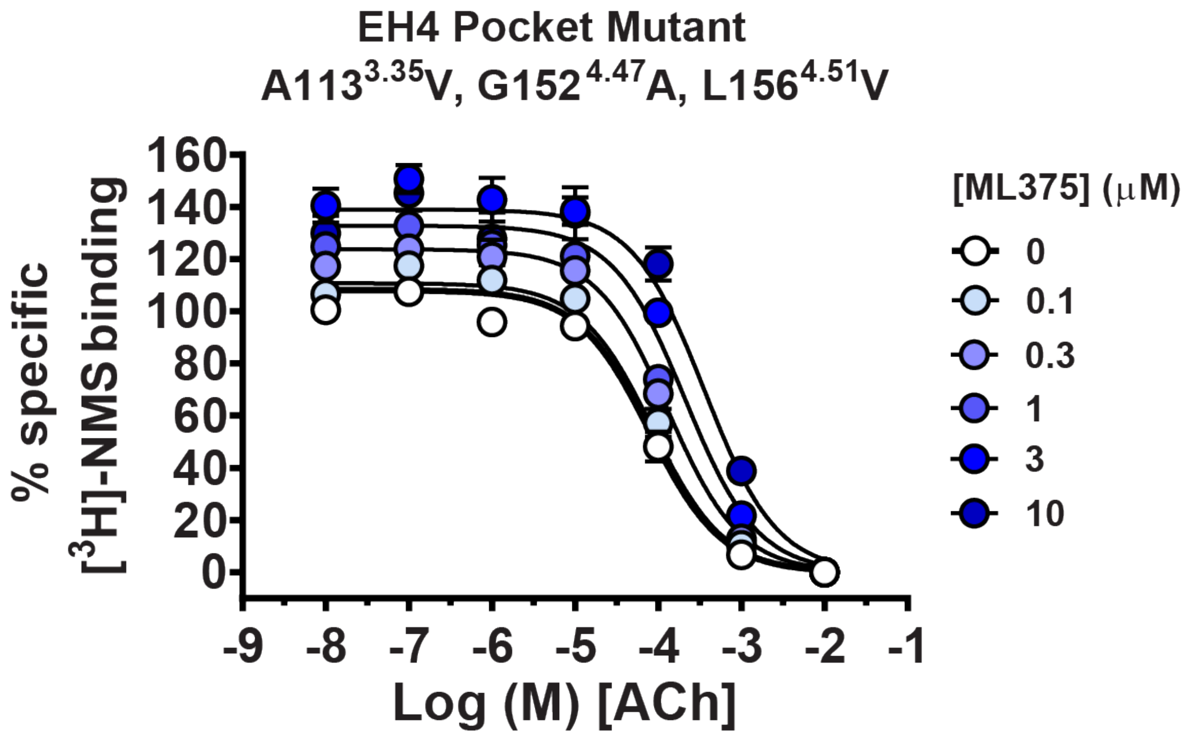

Supplementary Figure S4. ML375 binding at EH4 pocket mutant. Interaction between ACh, [^{3}H]-NMS and ML375 at the EH4 mutant expressed in FlpIn CHO cells. Data represent the mean ± S.E.M. of four independent experiments performed in duplicate. Parameters obtained in these experiments are listed in Table 2.

Supplementary Table 1 | Allosteric model parameters from radioligand binding assays for the interaction between ACh and ML375.

	[³H]-NMS Saturation Binding		Interaction binding between [³H]-NMS and Ach in the presence of ML375			
**Constructs**	[a]pK_D[1]	[b]B_{max} (fmol/10^5 cells)[1]	[c]pKi (ACh)	[d]Log α (ACh)	[e]pKb	[f]Log α (NMS)
**M2 WT**	9.6 ± 0.2 (4)	12.8 ± 2.9 (4)	5.4 ± 0.1 (6)	N.D.	N.D.	N.D.
**M2–M5-ECL1 (transient)**	9.3 ± 0.1 (3)	4.6 ± 1.2# (3)	4.7 ± 0.1* (3)	N.D.	N.D.	N.D.
**M2–M5-ECL2**	9.3 ± 0.1 (3)	13.0 ± 0.6 (3)	5.7 ± 0.1* (4)	N.D.	N.D.	N.D.
**M2–M5-ECL3**	10.0 ± 0.03 (4)	14.3 ± 1.8 (4)	5.9 ± 0.2* (4)	N.D.	N.D.	N.D.
**M2–M5-all-ECLs**	9.4 ± 0.04 (3)	18 ± 2.0 (3)	5.8 ± 0.2* (4)	N.D.	N.D.	N.D.
**M5 WT**	9.4 ± 0.2 (4)	13.8 ± 1.2 (4)	5.0 ± 0.1 (7)	-1.6 ± 0.2 (7)	6.9 ± 0.2 (7)	-0.06 ± 0.02 (7)
**M5–M2-ECL1**	9.3 ± 0.02 (3)	17.0 ± 0.6 (3)	4.6 ± 0.1* (4)	-1.2 ± 0.1* (4)	6.6 ± 0.1 (4)	0.2 ± 0.04* (4)
**M5–M2-ECL2**	9.7 ± 0.02 (3)	7.3 ± 0.7* (3)	4.7 ± 0.1* (3)	-1.4 ± 0.1 (3)	6.9 ± 0.1 (3)	-0.1 ± 0.02 (3)
**M5–M2-ECL3**	9.7 ± 0.1 (5)	8.4 ± 1.3* (5)	5.1 ± 0.1 (4)	-1.6 ± 0.2 (4)	6.9 ± 0.2 (4)	-0.08 ± 0.04 (4)
**M5–M2-all-ECLs (transient)**	9.0 ± 0.1 (3)	3.0 ± 0.4# (3)	4.8 ± 0.1* (4)	-1.2 ± 0.2* (4)	6.8 ± 0.3 (4)	-0.08 ± 0.03 (4)

Data represent the mean ± S.E.M. of (n) independent experiments performed in duplicate. N.D. Not determined *significantly different from WT, $p < 0.05$, one-way ANOVA, Dunnett's post hoc test. # Constructs were tested using transient transfections, hence the comparatively lower expression values.

[a] Negative logarithm of the radioligand equilibrium dissociation constant.

[b] Maximum density of binding sites.

[c] Negative logarithm of the orthosteric agonist equilibrium dissociation constant.

[d] Logarithm of affinity cooperativity between orthosteric agonist and allosteric modulator.

[e] Negative logarithm of the allosteric modulator equilibrium dissociation constant.

[f] Logarithm of affinity cooperativity between [³H]-NMS and allosteric modulator.

[1] Data Reproduced from Vuckovic *et al*.

Pocket	Docking Score
ECV	-26.1
EH1	N/A
EH2	-18.6
EH3	-19.6
EH4	-19.4
EH5	-22.6
IC1	-19.25

Supplementary Table 2. Docking scores of ML375. Docking of ML375 into the pockets gave a wide range of scores. Docking scores for EH1 are not available as the top three poses for ML375 were located outside of the respective pocket.
